# Textiloma mimicking retroperitoneal sarcomatoid carcinoma: A case study of diagnostic and therapeutic challenges.

**DOI:** 10.1016/j.ijscr.2024.110290

**Published:** 2024-09-15

**Authors:** Salim Ouskri, Ahmed Ibrahimi, Hamza Elabidi, Iliass Bourekba, Hachem El Sayegh, Yassine Nouini

**Affiliations:** Ibn Sina University Hospital Center, Morocco

## Abstract

**Introduction:**

textilomas, caused by forgotten surgical sponges, triggers inflammatory reactions, leading to complications like abscesses and fistulas. Often asymptomatic or presenting as an abdominal mass, they pose diagnostic challenges. This case study reports a textiloma mimicking a retroperitoneal sacromatoid carcinoma, discovered post-tumorectomy, and explores related diagnostic and therapeutic issues.

**Case report:**

A 58-year-old woman with a history of ureterolithotomy presented with chronic lumbar pain, fever, and a right retroperitoneal mass. Clinical and biological examinations indicated an inflammatory syndrome. CT and MRI imaging revealed a well-defined cystic mass, suspected to be a sarcomatous tumor. Surgical removal of the mass followed oncological principles. Histopathological examination confirmed it was a textiloma, a reaction to a forgotten surgical sponge, with no malignancy.

**Discussion:**

a textiloma, or gossypiboma, is a rare postoperative complication caused by forgotten surgical sponges, is common in intraperitoneal and gynecological regions, it is rare retroperitoneally. Textilomas provoke acute inflammatory reactions leading to abscesses or chronic inflammation causing fibrosis and calcifications. Diagnosis is challenging due to non-specific clinical signs, often requiring radiological investigations. CT and MRI can reveal characteristic features like serpiginous centers and spongiform appearances. Surgical removal is necessary. Recovery is usually uncomplicated in 60 % of cases, though severe complications and deaths can occur in delayed interventions.

**Conclusion:**

The medical history and imaging are crucial for diagnosing textilomas, whose incidence has decreased with radio-opaque marked sponges. Despite this, meticulous sponge counting remains essential but insufficient to completely eliminate the risk.

## 1 Introduction

A textiloma, is a rare but documented postoperative complication resulting from the presence of forgotten surgical sponges or drapes. Its frequency is estimated at 1 in 1000 to 1500 surgical operations [2]. This frequency has decreased due to safety measures and medico-legal implications, but factors such as obesity, emergencies, intraoperative bleeding, and changes in procedure can reduce surgical vigilance. The presence of a foreign body causes an inflammatory reaction, with encapsulation and granuloma formation, or complications such as abscesses, fistulas, or migration through hollow organs. A textiloma may remain asymptomatic or present as local suppuration or an abdominal mass, leading to diagnostic errors. It is mainly found in the abdominopelvic region and is rare in the kidneys [2,3].

This article reports a case of a textiloma simulating a retroperitoneal sarcoma, discovered through histopathological examination after a tumorectomy, and discusses the diagnostic and therapeutic challenges of this iatrogenic pathology.

## 2 Case report

It's a 58-year-old woman with a history of ureterolithotomy via a subperitoneal approach for a right ureteral stone 20 years ago presented with chronic lumbar pain that appeared postoperatively and recurred with episodes of fever and painful exacerbations, empirically treated with antibiotics and analgesics.

Clinical examination found a patient who was afebrile with tenderness in the right lumbar fossa as well as the peri-umbilical region, and the presence of a hard, resistant mass in the same region. Biological examination revealed an inflammatory syndrome with a CRP of 50 mg/l and a white blood cell count of 11,000.

**Fig. 1:**
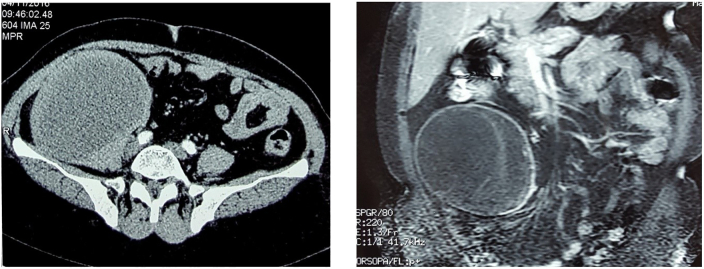
CT imaging showing the right retroperitoneal cystic mass.

CT imaging reveals a well-defined right retroperitoneal cystic mass, adjacent to the psoas muscle with which it appears to have partial continuity. The mass was however independent of the kidney, adrenal gland, and excretory system. Its content is relatively homogenous and hypodense, with peripheral enhancement following contrast administration (Fig. 1).

**Fig. 2:**
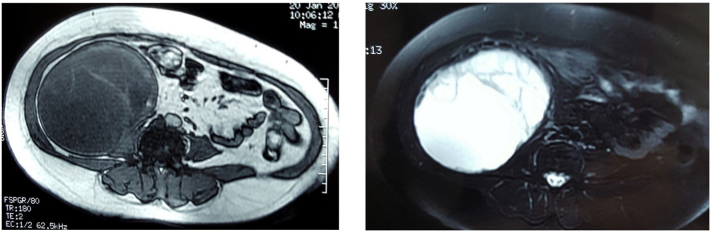
MRI confirming the cystic nature of the mass.

Subsequent MRI confirms the cystic nature of the mass, though it appears somewhat impure, exhibiting a heterogeneous T2 signal, with internal septations that are enhanced after gadolinium administration. No fatty components were observed (Fig. 2). The diagnosis considered by the radiologists was that of a sarcomatous mass, most likely an undifferentiated pleomorphic sarcoma.

Following these investigations, the patient underwent surgery through a lumbar incision below the 12th rib. The cystic mass was removed following oncological principles. The histopathological examination indicated a foreign body reaction suggestive of a *textilloma* with no signs of malignancy.

## 3 Discussion

A textiloma, or gossypiboma, is a rare but serious complication occurring after surgical interventions, with an estimated frequency of 1 to 3 per 10,000 operations. Primarily found in intraperitoneal and gynecological regions, it is exceptionally located retroperitoneally. The materials involved may include surgical sponges, gauze, or drapes, which provoke an acute inflammatory reaction in case of infection, often leading to abscess formation with alarming clinical signs necessitating immediate re-intervention. In the absence of infection, a chronic inflammation ensues, resulting in fibrosis, encapsulation, and calcifications, progressing insidiously and often discovered incidentally or presenting as a tumor syndrome [[2], [3], [4]].

Retroperitoneal textilomas are particularly difficult to diagnose due to their rarity and the absence of specific clinical signs. The natural history of a textiloma primarily depends on the body's reaction to the foreign object. Surgical sponges, being inert, do not decompose. Pathologically, two types of reactions can occur. The first is a sterile fibrous encapsulation reaction, leading to adhesion formation and granulomatous structures. This reaction is often asymptomatic, though a palpable mass may sometimes be observed. The second reaction is an exudative response, leading to abscess formation. This can result in peritonitis or fistula formation, causing patients to seek urgent care due to the severity of the symptoms. In both cases, clinical signs are not specific and can appear months or even years after the initial surgical intervention. Therefore, radiological investigations are essential in establishing the diagnosis before any surgical exploration [[3], [4], [5]].

The CT appearance of a textiloma often shows a well-defined rounded formation, with or without peripheral enhancement after contrast injection. The center of the lesion presents heterogeneous density, with hyperdens ribbon-like structures corresponding to the foreign object. In the study published by Choi and al. the serpiginous center is due to different stages of disintegration. Sometimes, air trapped between the fibers of a sponge is responsible for a spongiform appearance, gas bubbles can persist within the textile for a long time, and their presence does not predict the time interval after surgery. This aspect, however, is not often reported [2].

On MRI, they are generally well-defined rounded structures, with a low signal on T1 and a hyper signal on T2. Additionally, a serpiginous low signal structure in the center of the formation on T2 sequences is highly suggestive of retained sponges [2].

The differential diagnosis includes hematomas, abscesses, and cysts, and biopsy is often contraindicated due to the risk of malignant cell dissemination in case of cancer [2,4].

The treatment of a textiloma is always surgical, aiming to remove the foreign object to stop the inflammatory process. However, this intervention may be complex in advanced stages due to fibrosis and adhesions. The literature review shows that excision of the textiloma leads to uncomplicated recovery in nearly 60 % of patients, while severe complications occur in about 21 %, and nearly 19 % of patients die, often due to septic complications following delayed interventions [[4], [5], [6]].

## 4 Conclusion

The medical history plays a crucial role in diagnosis, complemented by imaging examinations for topographic evaluation and the search for complications. In the United States, the use of radio-opaque marked sponges since 1940 has significantly reduced these incidents, although counting sponges at the beginning and end of the procedure remains essential but still insufficient to completely eliminate this risk [3].

## 5 Methods

This work has been reported in line with the SCARE criteria.

## Consent

Written informed consent was obtained from the patient for publication and any accompanying images. A copy of the written consent is available for review by the Editor-in-Chief of this journal on request.

## Ethical approval

Ethical approval for this study was provided by the Ethical Committee of IBN SINA University Hospitals, Rabat, Morocco on 10/07/2024.

## Funding

NA.

## Author contribution

Salim Ouskri Urology Resident: FIRST author has contributed in the writing and correction the case report.

Ahmed IBRAHIMI Urology assistant Professor: SECOND author has contributed in the writing and correction the case report.

Hamza ELABIDI Urology Resident: has contributed in the correction the case report.

Iliass BOUREKBA Radiology resident: has contributed in the writing and correction the case report.

Imad Boualaoui Urology assistant Professor / has contributed in the correction the case report.

Hachem El Sayegh Urology Professor: has contributed in the correction the case report.

Yassine Nouini Urology Professor: has contributed in the correction the case report.

## Guarantor

Salim Ouskri.

## Research registration number

N/A.

## Conflict of interest statement

NA.
